# An Audit on Antidepressant Prescribing Practices for Children and Adolescents With Depression in Tonteg Hospital, Tonteg

**DOI:** 10.1192/bjo.2025.10608

**Published:** 2025-06-20

**Authors:** Amani Hassan, Chikwado Iwudibia

**Affiliations:** Cwm Taf Morgannwg University Health Board, Abercynon, United Kingdom

## Abstract

**Aims:** To measure the extent to which management of depression in children and adolescents compares with standard guidelines.

To enhance the quality of care and improve management practices for depression in children and adolescents.

**Methods:** Source of data: Electronic patient records.

Audit time frames:

Initial audit: 01/01/2017–31/01/2024.

Re-audit: 01/06/2024–31/01/2025.

Retrospective data.

Inclusion Criteria: Children and adolescents from the Taf Ely area within the Rhondda Cynon Taf Council, Wales diagnosed with depression and started on antidepressants between January 2017 and January 2025 in Tonteg Hospital were studied.

Exclusion Criteria: Patients prescribed antidepressant medication without a diagnosis of Depression.

**Results:** Demographics: In both the initial audit and the re-audit, females outnumbered males. The age range was 13–18 years.

Findings: In the initial audit, 82.6% (38/46) of the patients had other diagnosis (e.g., anxiety, eating disorders, PTSD). This was 54.5% (6/11) in the re-audit. There were no comorbid cases of bipolar disorder and psychosis.

Psychological therapy was provided to 63% (29/46) of patients before initiating antidepressants in the initial audit, improving to 82% (9/11) in the re-audit.

100% of the patients were prescribed a single antidepressant medication in the initial audit and re-audit. Fluoxetine and sertraline were the only prescribed antidepressants. No other psychotropic medication was prescribed.

**Conclusion:** The findings are not different with regards to the prevalence of depression in males compared with females. There is a higher prevalence of depression in females in both audit and re-audit.

In the re-audit, there is an 82% compliance with the latest NICE guidelines for the treatment of depression in children and adolescents. This is an improvement from the initial audit which showed a 63% compliance.

Following the initial audit, it was recommended that psychotherapy must be considered before starting any child with a diagnosis of depression on an antidepressant medication. Also, this information must be included in letters sent to the GP. These recommendations were effectively implemented, contributing to improved compliance in the re-audit.

